# Individual differences in emotion-induced blindness: Are they reliable and what do they measure?

**DOI:** 10.3758/s13414-024-02900-y

**Published:** 2024-05-17

**Authors:** Mark Edwards, David Denniston, Camryn Bariesheff, Nicholas J. Wyche, Stephanie C. Goodhew

**Affiliations:** School of Medicine and Psychology (Building 39), The Australian National University, Canberra, 2601 Australia

**Keywords:** Emotion, Attention, Emotion-induced blindness, Reliability, Measurement, Individual differences

## Abstract

The emotion-induced-blindness (EIB) paradigm has been extensively used to investigate attentional biases to emotionally salient stimuli. However, the low reliability of EIB scores (the difference in performance between the neutral and emotionally salient condition) limits the effectiveness of the paradigm for investigating individual differences. Here, across two studies, we investigated whether we could improve the reliability of EIB scores. In Experiment 1, we introduced a mid-intensity emotionally salient stimuli condition, with the goal of obtaining a wider range of EIB magnitudes to promote reliability. In Experiment 2, we sought to reduce the attentional oddball effect, so we created a modified EIB paradigm by removing the filler images. Neither of these approaches improved the reliability of the EIB scores. Reliability for the high- and mid-intensity EIB difference scores were low, while reliability of the scores for absolute performance (neutral, high-, and mid-intensity) were high and the scores were also highly correlated, even though overall performance in the emotionally salient conditions were significantly worse than in the neutral conditions. Given these results, we can conclude that while emotionally salient stimuli impair performance in the EIB task compared with the neutral condition, the strong correlation between the emotionally salient and neutral conditions means that while EIB can be used to investigate individual differences in attentional control, it is not selective to individual differences in attentional biases to emotionally salient stimuli.

Visual attention is important for selecting a subset of the items from our visual world for detailed and intensive processing (Carrasco, 2011). Attentional priority can be applied to items for a number of reasons, including because they are *emotionally salient*—that is, they depict or signal threat, punishment, or reward (Anderson, 2005; Lipp & Derakshan, 2005; Most et al., 2005, 2007; Pauli & Röder, 2008; Sutherland et al., 2017; Vuilleumier et al., 2002). In other words, there can be an attentional bias toward emotionally salient stimuli. There appear to be individual differences in the extent to which emotionally salient stimuli are prioritized (B. A. Anderson et al., 2011; Berggren & Derakshan, 2013; Delchau et al., 2022; Fox et al., 2002; Frischen et al., 2008; Jin et al., 2018; Mogg et al., 2004) such that individuals prone to anxiety or negative affect more broadly can show greater attentional biases. However, studies that have investigated this potential relationship have found mixed results (Bar-Haim et al., 2007; Kruijt et al., 2019; MacLeod et al., 2019; Mogg et al., 2008). These mixed findings could indicate that the relationship between the attentional prioritization of emotionally salient stimuli and these individual differences is weak or nonexistent, or it could indicate that there are problems with how this potential relationship is investigated. In this study we investigated the latter possibility, specifically, issues with the ability of the experimental measures used to reliably rank-order people in terms of the magnitude of their attentional bias (i.e., test–retest reliability of the measures).

When considering the allocation of attention, an important distinction is between *spatial* and *temporal* allocation of attention. Spatial attention refers to how attention is allocated to different spatial regions. This includes shifting the focus of attention to different locations and regulating the size of the breadth of attention (Chong & Treisman, 2005; Goodhew, 2020; Posner, 1980). Temporal allocation of attention refers to how it is allocated across time at the same location. While a number of experimental paradigms have be used to investigate spatial and temporal attentional biases to emotionally salient stimuli, two commonly used ones have been the dot-probe paradigm for spatial (MacLeod et al., 1986) and emotion-induced-blindness (EIB) paradigm for temporal (Goodhew & Edwards, 2022b; Most et al., 2005).

The dot-probe paradigm consists of the brief presentation of two spatially offset stimuli, one neutral and the other emotionally salient, which then disappear and are replaced by a target which appears in the location of one of those stimuli. The participants’ task is to either detect or identify this target. The measure of each participant’s attentional bias is taken as the extent to which responses are facilitated when the target appears in the location that was occupied by the emotionally salient stimulus compared with the neutral stimulus (Frewen et al., 2008; Kappenman et al., 2014; MacLeod et al., 1986). The difference in response time between these two trial types removes differences in individuals’ generic speed of response, instead isolating a measurement of the allocation of attention to the emotionally salient stimulus (Goodhew & Edwards, 2022b).

The EIB paradigm consists of a rapid serial-visual-presentation (RSVP) sequence, similar to the attentional-blink paradigm, except that a single target is used, and a task-irrelevant distractor, that is either a neutral or emotionally salient, precedes the target close in time (typically, two images before the target in the RSVP stream with each image typically being presented for 100 ms). The target is the same as the filler images in the RSVP sequence (typically landscape or city scenes) except that it has been rotated 90 degrees, and the task is to indicate the direction of the rotation (left or right). The EIB effect is that task performance (i.e., accuracy at identifying the target’s rotation) is worse when the target follows the emotionally salient distractor compared with the neutral distractor. It is assumed that this EIB effect is due to greater attentional engagement with the emotionally salient distractor, compared with the neutral one and the difference in performance between the neutral and the emotionally salient conditions is taken as the measure of EIB. The difference score is used, in part, to attempt to remove differences in non-attentional factors between the participants—for example, differences in their sensitivity to the briefly presented images (Goodhew & Edwards, 2022b; Most et al., 2005). It has been shown that performance on the dot-probe and EIB tasks do not correlate with each other, and that they each explain unique variance in participants’ self-report negative affect in everyday life (Onie & Most, 2017). Both of these findings indicate that they are tapping different aspects of attentional control, and hence, are consistent with them tapping spatial and temporal aspects of attentional deployment, respectively (Onie & Most, 2017).

One way to establish whether there is a relationship between a bias in spatial and/or temporal attention to emotionally salient stimuli and an individual difference variable such as negative affect is to determine whether there is a correlation between scores on a measure of attentional bias (e.g., the EIB task) and a scores on a measure of negative affect (e.g., the Depression, Anxiety, and Stress Scales [DASS-21]; Lovibond & Lovibond, 1995). However, the ability to find a relationship between two variables (if there is one to be found) is constrained by the rank-order reliability of the measures of each variable (Spearman, 1910). That is, when investigating the correlation between two variables, the maximum correlation that can be found is equal to the actual correlation divided by the square root of the product of the reliability of the two measures. Here, by reliability, we mean test–retest rank-order reliability: the ability to consistently rank-order people if they are tested multiple times using the same measure, or, if a single test is administered, that all of the scores in that test result in the same estimate of the person’s score. The latter is typically assessed using split-half analysis—for example, comparing the scores for the first half of the trials to those for the second half. However, how the individual trials are divided into halves can significantly affect the split-half reliability measure obtained, so it is best to base the estimate of reliability on many different split halves (Parsons et al., 2019). Only when the reliability of the two measures is perfect (i.e., their reliabilities equal 1) will the measured correlation equal the true correlation. This limitation makes logical sense given that the rank-ordering of the participants on, say, their EIB magnitude, is taken as a proxy for the rank-ordering of the magnitude of their temporal attentional-bias to emotionally salient stimuli. If there is variability in that rank ordering, such that it is not consistent across repeated trials, it (potentially) means that we do not have a stable measure of the attentional bias and hence we cannot determine its relationship with another factor. There are two important caveats to this point. The first is that a reliable measure does not ensure that the measure is valid. For example, taking a person’s height as a measure of attentional bias will result in a stable measure in adults and hence result in reliable rank-ordering, but it would not be a valid measure of their attentional bias. Secondly, the lack of reliability in the bias measure may be a legitimate finding. That is, the attentional bias may vary over time, depending upon factors like the person’s emotional state and the situation they are in, rather than a stable trait variable (Cox et al., 2018; Zvielli et al., 2015).

Altogether, this means that being able to both measure the magnitude of attentional bias and being able to reliably rank-order people in terms of that magnitude (if the attentional bias is stable over time) is of fundamental importance to studying the potential existence, nature, and consequences of any relationship between the attentional bias and other individual difference factors, such as negative affect. Unfortunately, neither the standard dot-probe nor the EIB paradigms have good rank-order reliability (Onie & Most, 2017; Schmukle, 2005). Studies have been devoted to trying to improve the reliability of the dot probe paradigm (Carlson & Fang, 2020; Chapman et al., 2019; Zvielli et al., 2016), but thus far, to the best of our knowledge, this is not the case for the EIB paradigm. One study that explicitly documented the reliability of EIB indicated its intraclass correlation coefficient was 0.42 for the difference score (Onie & Most, 2017). This compares to a recommended reliability benchmark of at least 0.7 for individual-difference research (Hedge et al., 2018). Therefore, the focus of the present study was on attempting to improve the measurement reliability of attentional bias as gauged by EIB.

This poor reliability in the EIB measure likely contributes to the inconsistent findings in the literature regarding how individual differences in EIB scores vary with a range of individual difference factors. For example, while Onie and Most (2017) found a relationship between EIB and negative affect as measured by a hybrid measure of the DASS, the Penn State Worry Questionnaire, and the Rumination Response Scale (thought note, they did not find this relationship with the standard EIB difference score, but only for absolute performance following the negative emotionally salient distractors), another study by Most and colleagues failed to find such a relationship, though this time just using the DASS as the measure of negative affect (Guilbert et al., 2020). This lack of a relationship between EIB and DASS is consistent with the findings of studies by other researchers (Kennedy et al., 2020; Perone et al., 2020). Note also that the study by Kennedy and colleagues tested the relationship for both the standard EIB difference scores and the absolute scores. Similarly for anxiety, one study has found a relationship (Chen et al., 2020) while another has not (Kennedy & Most, 2015) and for harm avoidance, one has found a relationship (Most et al., 2005), while another has not (Most et al., 2006). These mixed findings are consistent with our experiences, sometimes we have found a relationship between EIB scores and DASS, while at other times we have not (unpublished data) and it was these inconsistencies that prompted us to consider the reliability of EIB scores and to explore whether their low reliability could be contributing to these mixed findings in the literature.

How then might we improve the reliability of the EIB paradigm? Hedge et al. (2018) have noted that there is a fundamental difference between the requirements for measures developed for experimental research and those developed for individual-differences research, when it comes to the ideal variance in performance between individuals on the measure. With experimental research, the aim is to achieve the greatest experimental effect, and this is typically achieved by all participants showing the greatest effect for the experimental manipulation of interest and hence scoring similarly on the outcome measure of interest. However, the opposite is true for individual difference research. There, the aim is to be able to detect differences in the measure of interest between participants and to be able to reliably rank-order participants on those differences. If all individuals score similarly on the measure of interest, then rank ordering people on differences is not possible. Having greater variance in the magnitude of the experimental effect across participants on a given task will increase the likelihood of being able to reliably rank order them—as long as that variance in performance actually reflects stable variation in the process of interest (Goodhew & Edwards, 2019; Hedge et al., 2018). This means that all other things being equal, paradigms where an experimental manipulation has a more modest effect when averaged across participants due to increased variability in participant scores may actually be more suited to the goals of individual differences research than one where there is a larger magnitude effect when averaged across participants.

Given this difference in requirements for experimental and individual research (minimizing versus maximizing between-participant variance), experimental paradigms that have been optimized for experimental studies are unlikely to work well in individual difference studies. Arguably, this is the case with the EIB paradigm. In order to elicit a strong EIB effect at the group level, the intensity of the emotionally salient stimuli used are maximized. That is, emotionally salient stimuli are used that have extreme values on both valence and arousal. This means that they are likely to be highly emotionally salient for most if not all participants, regardless of the strength of their attentional bias to emotionally salient stimuli, resulting in a strong EIB effect for (most) participants and hence minimizing variance in EIB magnitude between participants. This is typically achieved by using stimuli from the International Affective Picture System (IAPS; Lang et al., 2008) that are rated as intensively negative (on the dimension of valence) and highly arousing (on the dimension of arousal; Goodhew & Edwards, 2022b). Thus, one logical way to potentially increase between-participant variability of EIB magnitudes is to reduce the intensity of the images used (Goodhew & Edwards, 2019). This can be explained via Fig. 1, which shows how we propose EIB magnitude would vary as a function of the intensity of the emotionally salient stimuli for people with three different attentional-bias strengths. EIB magnitude is plotted against the intensity of the emotional distractor. Images with the highest emotional intensity would result in a large EIB magnitude for all three strengths of attentional bias, hence those images cannot differentiate participants who have different attentional-bias strengths. Low intensity images result in the same inability to differentiate between the different attentional biases, but now because all participants would exhibit the same, low EIB magnitude. However, if medium-intensity stimuli were used, then we predict that participants with different strengths of attentional bias would exhibit different EIB magnitudes.

**Fig. 1:**
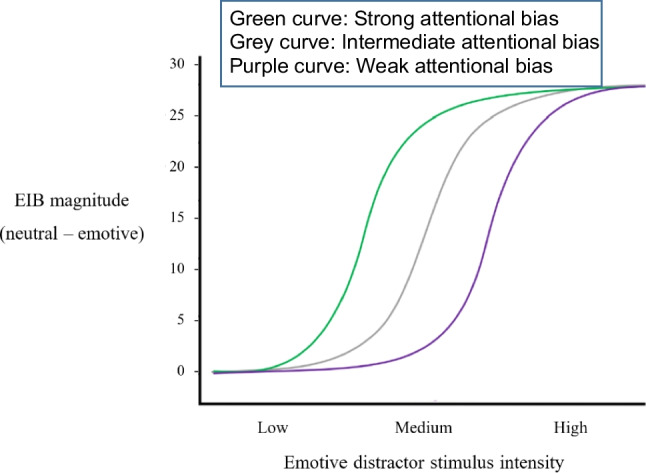
An illustration of how an intermediate stimulus intensity may be optimal for distinguishing different individual levels of attentional bias. *Note.* EIB magnitude (the difference in performance between the neutral and emotive conditions) (*y*-axis) is plotted against the intensity of the emotionally salient distractors (*x*-axis) and hypothetical psychometric functions are shown for three different strengths of attentional bias to emotionally salient stimuli. The green curve represents a strong attentional bias, the purple curve a weak bias, and the grey curve an intermediate bias. As can be seen, when high-intensity images are used, there is little to no difference in the EIB magnitude for the three different attentional-bias magnitudes. The same would be true if low-intensity stimuli were used. However, if medium-intensity stimuli are used, we predict that differences in EIB magnitude would be obtained. It should be noted that these labels of low, medium, and high for the intensity of the emotional stimuli would map onto the IAPS ratings of arousal and valence ratings for the stimuli. These ratings are the average responses from many people and are ranked on a 1 to 9 scale. For arousal, the scale goes from low (1) to high (9) and for valence very negative (1), through neutral (5), to very positive (9). For negative images, Low emotionally salient would be near-neutral valence and low arousal, medium would be more negative in valence and higher in arousal, while high would be the most negative in valence and highest in arousal. (Colour figure online)

Thus, in our first study, we determined the reliability of the EIB measure for both the standard high-intensity stimuli and for mid-intensity stimuli. We predicted that we would obtain greater meaningful variance and hence greater reliability for the mid-intensity condition.

## Experiment 1: Mid-intensity emotionally salient stimuli

### Method

#### Participants

Sample size was determined by a power analysis, using G*Power’s point biserial function (Faul et al., 2009) and a medium effect size, (*r* = .30). Onie and Most (2017) observed slightly above a medium effect size relationship between EIB and negative affect (*r* = −.32), and thus assuming a medium effect is a conservative approach to ensuring sufficient power. This indicated a sample of 134 required to have 95% power of detecting a medium effect with a two-tailed test. Given that in previous EIB studies conducted in our lab we have had exclusion rates of up to 15% for failure to comply with task instructions, we recruited 154 participants from the general public and the Australian National University (ANU) community (male *N* = 80 [51.95%], female *N* = 72 [46.75%], other *N* = 2 [1.3%], *M =* 24.00 years, *SD* = 7.41). All reported having normal or corrected-to-normal vision, were over 18 years of age, and were offered either AUD$15 or research participation course credit for their time. Participants gave voluntary, informed consent, and the study was approved by the ANU Human Research Ethics Committee under Protocol 2018/633.

#### Apparatus and stimuli

The EIB paradigm requires four types of images: filler images, target images, emotionally salient distractors, and emotionally neutral distractors. Filler images were 290 landscape and architectural pictures which we have used in a previous study (Proud et al., 2020). The filler images did not contain people, animals, household items, or abstract art to ensure that they were distinct from the distractor images. Target images consisted of 240 images that were from the same categories as the filler images (landscapes and architectural pictures), except that they were rotated either left or right by 90°.

The emotionally salient and emotionally neutral distractors were IAPS images and were selected based upon their arousal and valence ratings from men and women in the IAPS database. Three distractor conditions were used: neutral, high-intensity emotionally salient, and mid-intensity emotionally salient. The mean arousal and valence ratings for each of these conditions are given in Table 1. Each of these distractor sets contained 60 images. The neutral images depicted household items, flora, fauna abstract art, or people in everyday situations; the high-intensity images depicted mutilated bodies, violence, guns pointed directly at the camera, or disgusting scenes; and the mid-intensity images people in dangerous or uncomfortable situations, expressions of mild-to-moderate discontent, animals poised to attack, weapons portrayed in otherwise nonthreatening contexts, or messy or dirty areas.

**Table 1:** Means and standard deviations for the IAPS normative arousal and valence ratings for the stimuli in the three distractor conditions

Condition	Arousal rating	Valence rating
**Neutral**	3.56 (0.58)	5.29 (0.13)
**Mid-intensity**	5.23 (0.82)	3.57 (0.25)
**High-intensity**	6.45 (0.55)	1.91 (0.31)

*Note.* Arousal ratings ranged from 1 (*low arousal*) to 9 (*high arousal*) and valence ranged from 1 (*very negative*), 5 (*neutral*), to 9 (*very positive*). See Appendix 1 for a list of the actual images used and their arousal and valence ratings

Stimuli were presented in a uniformly lit room on a liquid-crystal-display monitor that had a spatial resolution of 1,920 × 1,080 pixels and temporal refresh-rate of 60 Hz. Participants were seated and a chinrest was used to maintain a viewing distance of 550 mm to the screen. The images were presented on a white background and their visual angle was 11.2 degrees high by 18.4 wide. Stimuli were presented using code that was custom written using MATLAB’s Psychophysics Toolbox extension. Individual differences in negative affect were measured using the short form version of the Depression, Anxiety and Stress Scales (DASS), the DASS-21 (Lovidbond & Lovibond, 1995). Here we used DASS-21 total score, which has been shown to be a valid gauge of general negative affect (Henry & Crawford, 2005) and has been used in previous research examining correlates of emotion-induced blindness (e.g., Onie & Most, 2017). It consists of 21 statements (e.g., “I found it difficult to relax”) for which participants select one of four response options: Never (0); Sometimes (1); Often (2); Almost Always (3), to indicate how often these statements applied to them over the past week. DASS-21 total score is calculated by summing the response values and multiplying them by two (to make them comparable to the DASS-42 range). Thus, possible scores range from 0 to 126, where higher scores indicate greater negative affect. The DASS-21 was administered via Qualtrics. One attention-check item was included that instructed participants to select the response “Almost Always.”

#### Procedure and design

Each experimental trial commenced with a central fixation cross that was presented for 500 ms, followed by a 17-image EIB sequence in which each image was presented for 100 ms. At the end of the sequence a blank screen was presented until the participant made their response as to the orientation of the target (via the computer keyboard’s left or right arrow keys). Each EIB sequence contained 15 fillers, one distractor and one target. The temporal position of the distractor was randomized to fall in Position 2, 4, 6, or 8 in the sequence. The target appeared in Lag 2, that is, it was the second image after the distractor.

The experiment commenced with 15 practice trials. These were the same as the experimental trials except that long (3,000 ms) image durations were used for the initial trials, with the duration being progressively shortened until it was the experimental duration (100 ms) for the final trials, and onscreen feedback on the accuracy of each response was given after each response. Additionally, only neutral distractors were used. Participants progressed to the experimental blocks once they achieved a performance level of 80% or more on the practice trials that were presented for 100 ms. If they could not achieve that level after 15 minutes of testing, the experiment was terminated.

In the experimental trials, each distractor image was shown twice, resulting in 120 trials for each distractor condition. Trial types (i.e., high intensity, mid intensity, or neutral distractor) were randomly intermixed. At the end of the experimental trials, participants were shown a sequence of 20 pleasant images (e.g., images of kittens and puppies) each presented for 3,000 ms, to help mitigate any lingering effects of the negative experimental images.

### Results

#### Data screening

De-identified raw data are available here: (https://osf.io/sgebu/). Data analysis was performed in JASP (JASP Team, 2020). Of the 154 participants, nine were excluded from the final analysis due to failing the performance cut-off. Given that chance performance was 50%, we used a performance cut-off of less than 60% in the Neutral condition as an indicator of the participant being either unwilling or unable to perform the task. This resulted in a final sample of 145, composed of 50.3% male, 48.3% female, 1.4% other, mean age 23.97 years (*SD* = 7.46).

#### EIB performance

We first investigated EIB performance for the three EIB conditions at the group level. The average performance levels are shown in Table 2. As expected, performance was highest for the neutral condition, followed by the mid- and then high-intensity conditions. The data were normally distributed (skew and kurtosis *z*-score absolute values <2.58), so to test these differences we used a repeated-measures analysis of variance (ANOVA), which indicated a significant difference, *F*(2, 288) = 230.04, *p* < .001, η_p_^2^ = .62. Paired-sample *t* tests at a Bonferroni-corrected *p* = .017 showed that the means for all conditions differed from each other (*p*s < .001, Cohen’s |*d*s| > .91).

**Table 2:** Means and standard deviations for percent-correct for the three EIB conditions used in Experiment 1

Condition	Mean	Standard deviation
**Neutral**	77.81	7.20
**Mid intensity**	72.59	8.15
**High intensity**	66.63	7.45

Next, we calculated the standard, group EIB magnitudes (averaged across participants). That is, mean differences in performance between the neutral condition and each of the high- and mid-intensity conditions. The high-intensity condition had an EIB magnitude of 11.16% (*SD* = 6.78) and the mid-intensity condition 5.22% (*SD* = 5.71). The EIB scores were normally distributed. A paired *t* test showed that the EIB magnitude for the high-intensity condition was greater than that for the mid-intensity condition, *t*(144) = 11.36, *p* < .001, Cohen’s *d* = .94, 95% CI for Cohen’s *d* [.75, 1.14].

#### Individual variation in performance and reliability

As a starting point, we investigated the correlation in performance between the three conditions. Using a Bonferroni-corrected significance level of *p* < .017, significant correlations were observed between all three conditions (see Table 3).

**Table 3:** Pearson’s *r* correlations between the three EIB conditions in Experiment 1

Comparison	Correlation	95% confidence intervals
**Neutral–High-intensity**	.57^***^	.45, .67
**Neutral–Mid-intensity**	.73^***^	.64, .80
**High-intensity–Mid-intensity**	.68^***^	.58, .76

*Note*. *** indicates *p* < .001

Next, we used split-half analyses to estimate rank-order reliability of the individual EIB magnitudes. Split-half analysis involves dividing the data for each participant in half and then determining how consistent the ranking is between the two halves by calculating the correlation between them. Given that the actual halves the data are divided into can significantly affect the correlation obtained, we ran a large number (5,000) of permutations, as recommended by Parsons and colleagues (Parsons et al., 2019) and used their split-half package for R, in order to calculate an accurate estimate of reliability (Parsons, 2020). The reliability scores for the two EIB magnitudes (i.e., difference scores), as well as the reliabilities of performance scores for the three distractor conditions are shown in Table 4. Reliability of both EIB difference scores were poor. Indeed, the confidence intervals for the mid-intensity’s condition reliability overlapped zero. These values stand in contrast to the reliability of the performance scores on the three distractor conditions raw scores (i.e., not difference scores), especially those for the neutral and mid-intensity conditions which are at or above the 0.7 value recommended for individual-difference research (Hedge et al., 2018).

**Table 4:** Reliability scores for the two EIB magnitudes and performance on the three distractor conditions in Experiment 1

Condition	Split-half reliability	95% confidence interval
**High-intensity EIB**	.29	.11 to .45
**Mid-intensity EIB**	.07	−.15 to .27
**Neutral distractor**	.74	.68 to .80
**High-intensity distractor**	.67	.59 to .74
**Mid-intensity distractor**	.76	.70 to .81

*Note.* The EIB scores are difference scores (accuracy in the named intensity minus neutral), whereas the other scores are the absolute scores from which the difference scores are calculated

#### Relationship between EIB and DASS-21

One additional participant was excluded from this analysis because they did not answer all of the DASS questions. DASS scores had a mean of 32.83 (*SD* = 16.61) which is comparable with those previously observed in university populations (Crawford & Henry, 2003). All distributions were normally distributed (skew and kurtosis *z-*score absolute values <2.58), so Pearson’s correlations were used to assess the relationships between EIB scores and DASS-21 scores. There were no significant correlations with DASS-21, with Pearson’s correlations of *r*(143) < .01, *p* = .982, 95% CI [−.16, .17] for the high-intensity condition and *r*(142) = −.04, *p* = .615 [−.20, .12] for the mid-intensity condition. In addition, absolute performance on the individual conditions were also not correlated with DASS-21 scores, *r*(143) = .08, *p* = .348 [−.09, .24] for neutral, *r*(143) = .07, *p* = .377 [−.09, .24] for high- and *r*(143) = .10, *p* = .237 [−.06, .26] for mid-intensity conditions.

### Discussion

The mid-intensity EIB condition did not result in greater split-half reliability than the high-intensity condition when the standard EIB magnitudes (i.e., difference scores) were used. Contrary to expectation, it actually resulted in numerically worse reliability (0.07 compared with 0.29). The reliability of both were well below the minimum value of 0.7 recommended for individual-differences research (Hedge et al., 2018). The reliability we observed in the standard high-intensity condition is similar to that obtained by Onie and Most (2017). They obtained a reliability estimate of 0.4 for EIB difference scores, which falls within the confidence interval around our estimate (which does not overlap zero). Therefore, the two studies converge in obtaining significant reliability for EIB scores, but reliability that is still well below acceptable levels for individual differences research.

Not surprisingly, given the low reliabilities of the EIB magnitudes, there was no significant relationship between EIB magnitude and DASS-21 for either condition (recall that low reliability limits the ability to find a relationship between two factors even if there is one to be found).

These low reliabilities stand in contrast to the reliabilities for absolute performance on the individual distractor conditions, and in particular the neutral and mid-intensity conditions. This pattern of results has been observed previously for the standard (high-intensity) EIB condition (Onie & Most, 2017) and is a well understood aspect of difference scores (Edwards, 2001). EIB is quantified using a difference score (performance in the neutral condition minus that for the emotionally salient condition) in order to remove generic perceptual and cognitive factors that are not related to the attentional bias to emotionally salient stimuli, like, for example, ability to resolve rapidly presented stimuli (Goodhew & Edwards, 2022b). However, the marked reduction in reliability in going from the absolute scores to the different scores would seem to indicate that what is driving the individual variation in performance for the neutral condition is also significantly driving it in the emotionally salient conditions. This idea is supported by the large-magnitude correlations between performance in the neutral condition and the two emotionally salient conditions (refer to Table 3).

The neutral condition is often thought of as a baseline, or reference condition, however, as noted by Hoffman and colleagues (Hoffman et al., 2020) performance on the neutral condition in EIB studies is significantly worse than that on a true baseline condition (i.e., one in which the distractor is replaced with another filler image). As they note, the images in the neutral condition (e.g., household appliances, flora and people in everyday situations) are physically distinct from the filler images (landscapes and cityscapes) and this physical salience could result in an oddball effect which could make them attentionally salient. That is, they could capture attention. More recent studies have provided further support to the idea that it is the physical salience of the distractors, compared with the filler images, that causes the initial attentional engagement with the distractor images (Baker et al., 2021; Santacroce et al., 2023). If this idea is correct, it would mean that performance in the neutral condition is, at least in part, affected by attentional control; specifically, the ability to stop attention being allocated to a physically salient but task-irrelevant image in a sequence. This attentional-control aspect in the performance for the neutral condition may account for the high correlation between the neutral and emotionally salient conditions, and hence the marked reduction in the reliability of the EIB scores that are taking the difference between those two scores (since, by taking the difference, the individual differences in performance are removed).

If the high correlation between the two conditions is being substantially driven by attentional control aspects underpinning performance in both conditions, what could this mean? The first possibility is that the pattern of individual variation in attentional control is the same for both emotionally neutral and emotionally salient stimuli. That is, the absolute magnitude of the attentional focus on the emotionally neutral and salient stimuli differs (see Table 3), but the pattern of individual variation is the same for both. A second possibility is that attentional control for emotionally salient stimuli is different to that for emotionally neutral stimuli, and that the correlation between the conditions is due to the emotionally salient images also being physically salient, by virtue of being different from the filler images. That is, there are two aspects of the emotionally salient images that are driving attentional allocation to them: their emotional salience and their physical salience, and the correlation between the neutral and emotionally salient conditions is being substantially driven by the physical salience aspect. If this latter possibility is the case, then in order to have a clean measure of a person’s attentional bias to emotionally salient stimuli, we need to remove the physical salience aspect from the experimental paradigm. That was the aim of the next experiment.

## Experiment 2: Removing the oddball effect of the distractors

The aim of Experiment 2 was to remove the oddball effect of the distractors. As noted by Hoffman and colleagues (Hoffman et al. 2020), having a series of filler images that are all similar and distinct from the distractors results in an attentional pop-out effect for the distractors, and this would contribute to the observed impairment in both distractors conditions in the EIB paradigm. The simplest way to achieve this would be to entirely remove the filler images, leaving just the distractor and the target images. This approach is similar to another experimental paradigm that we have developed, which we called emotion induced slowing (EIS). That stimulus consists of a sequence of two images: an IAPS image and a test image (Goodhew & Edwards, 2022a). Here, given that an accuracy measure was used, rather than reaction time, we also included masks after the IAPS and target images to limit the effective duration of the stimuli so as to be able to achieve the desired performance levels. No longer having the long RSVP sequence primarily composed of filler images means that there will be no oddball effect with the distractors due to their physical distinctiveness compared with the filler images. Note that the mask is presented after the distractor, so an oddball effect for the distractor relative to the mask is not produced. Given that this approach allows for the use of the standard neutral and emotionally salient IAPS sourced images as distractors, as well as the standard target images, this is the approach we used. We call this experimental paradigm emotion-induced-blindness-no-fillers (EIBNF). While this approach removes the oddball effect, it is not possible to entirely remove the physical salience difference between the distractor and the other images given that they are fundamentally different images. So, those differences will always remain.

### Method

#### Participants

Sample size was once again determined by a power analysis, using G*Power’s point biserial function (Faul et al., 2009). The effect size was assumed to be .30 as per Experiment 1 (but note that if power is based on the EIB difference score reliability estimate observed in Experiment 1, the assumed effect size is almost identical, *r* = .29, and we ultimately exceed the required *N* = 88 for that effect size). For practical reasons, the power level was set to 80% for this experiment. All other aspects of the power analysis were the same as Experiment 1, indicating a required sample of 82 participants. Assuming up to a 15% exclusion rate, we sought to recruit *N* = 94 to ensure that we met the minimum of *N* = 82 after exclusion.

#### Apparatus and stimuli

The same three distractor conditions and images from Experiment 1 were used in Experiment 2: neutral, high-intensity, and low-intensity emotionally salient. The same target images were used as in Experiment 1, and there were no filler images. In order to limit the effective presentation duration of each image, mask images were also used and presented after each image. The use of a mask stops the preceding image persisting in iconic memory (Coltheart, 1980). In a typical RSVP stream the subsequent filler items create this masking effect, here we had to create it without these items. This is particularly important for the target stimulus. If a mask did not follow the target stimulus, then the persistence of the target would make the task too easy resulting in ceiling level performance. Masks are most effective when they activate the same visual cells that process the experimental images (Breitmeyer, 1984; Tovée, 1998), so given the types of images that comprise the neutral, emotionally salient and target conditions, we developed masks composed of multiple objects of various sizes, shapes and colours. Ten masks were created, and 10 additional ones were created by flipping them about a horizontal axis, resulting in a total of 20 masks.

**Fig. 2:**
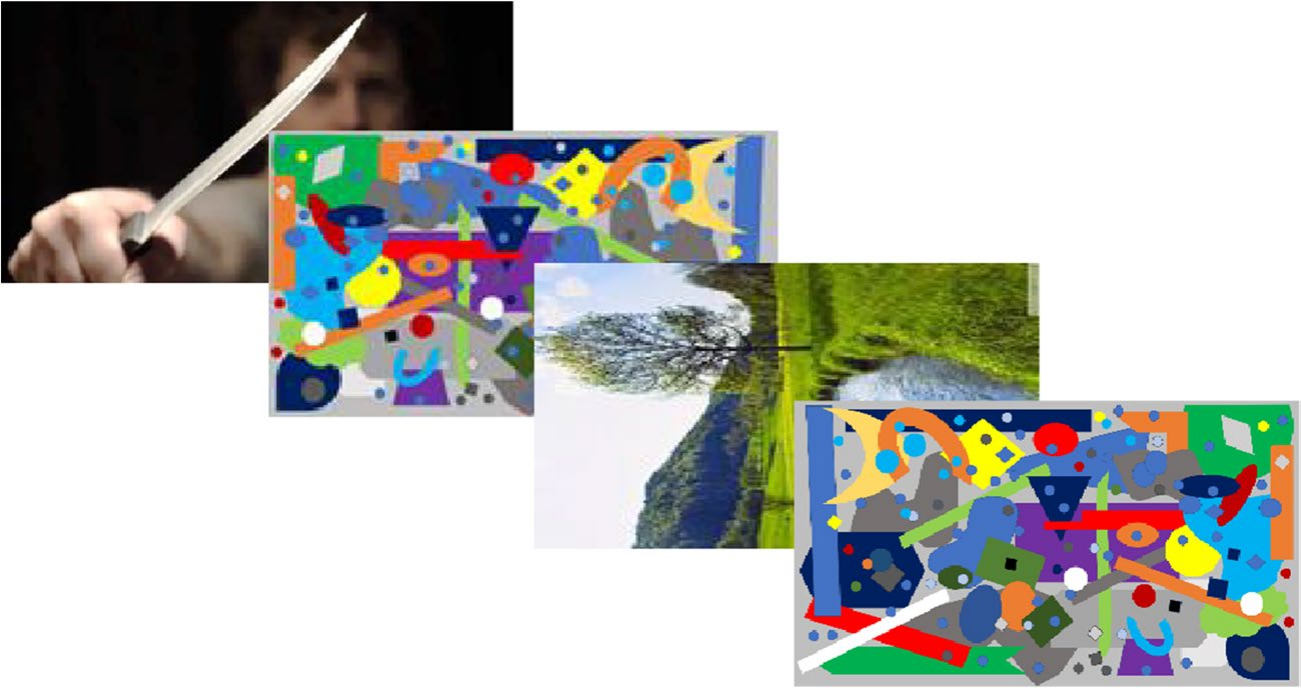
Schematic of the four-image EIBNF sequence used in each trial in Experiment 2. The first image depicts an emotionally salient distractor, the third image the target (rotated to the left), and images two and three are masks. The distractor was presented for 200 ms and the other three images for 100 ms each. (Colour figure online)

The trial sequence is depicted in Fig. 2. Each trial started with the presentation of a fixation dot (not shown here) that was presented for between 500 and 800 ms, followed by a four-image EIBNF sequence. The first image was the distractor (neutral, high intensity, or mid intensity) and the third image was the target. Images two and four were masks. At the end of the four-image sequence another fixation dot was presented (not shown here) until the participant responded.

#### Procedure and design

Each EIBNF trial began with the presentation of a fixation dot whose duration varied between 500 and 800 ms. This variable duration was used to minimize temporal learning effects, in which participants learn when a distractor appears which can then minimize the impact of that distractor (Geng et al., 2019). The fixation dot was followed by four-image EIBNF sequence. We pilot tested the durations of the stimuli in order to maximize the sensitivity of the paradigm. That is, to try to achieve performance for the neutral condition to around the 75% accuracy level (Goodhew & Edwards, 2019). This resulted in durations of 200 ms for the distractor and 100 ms each for the masks and the target. At the end of this sequence another fixation dot was presented until the participant responded. As per Experiment 1, at the end of the EIBNF block, participants were shown the positive-image block and the DASS-21 was administered.

### Results

#### Data screening

Raw data for this experiment are available online (https://osf.io/sgebu/). Of the 94 participants, three were excluded from the final analysis due to failing the performance cut-off (less than 60% accuracy in the neutral condition). This left a final sample of 91, which was in excess of the required 82. The sample comprised 62.6% female, 31.9% male, and 5.5% other. Mean age was 23.29 years (*SD* = 5.16).

#### EIB performance

We first investigated EIB performance for the three EIBNF conditions at the group level. The average performance levels are shown in Table 5. As expected, performance was highest for the neutral condition, followed by the mid- and then high-intensity conditions. All three distributions were not normally distributed (skew and kurtosis *z*-score absolute values >2.58), so a Friedman test was used to investigate whether there were differences among the means. This resulted in a significant difference, χ^2^(2) = 76.91, *p* < .001, and so a Conover test was performed on each of the pairs of conditions (Bonferroni-corrected level of *p* = .017). This indicated significant differences between the high-intensity condition and both the neutral and mid-intensity conditions, *t*(180) = 8.43, *p* < .001, and *t*(180) = 6.31, *p* < .001, respectively, but not between the neutral and mid-intensity conditions, *t*(180) = 2.12, *p* = .036. This means that at the group level, there was the standard EIB type effect for the high-intensity condition (i.e., significant difference between it and the neutral condition) but not for the mid-intensity condition. This, by itself, does not rule the mid-intensity condition out from being a potentially more reliable measure of the attentional bias to emotionally salient stimuli. This is because the logic of reducing the intensity of the emotionally salient stimuli is to not to have all participants show a strong attentional bias to the images, which would reduce the group level magnitude of the effect.

**Table 5:** Means and standard deviations of percent-correct for the three EIBNF conditions used in Experiment 2

Condition	Mean	Standard deviation
**Neutral**	88.78	8.67
**Mid intensity**	87.06	10.04
**High intensity**	82.71	10.70

#### Individual variation in performance and reliability

Our starting point was again to determine the correlation in performance between the three conditions. Given the data was not normally distributed, we used Spearman’s rho correlations. Using a Bonferroni-corrected significance level of *p* < .017, significant correlations were observed between all three conditions (see Table 6).

**Table 6:** Spearman’s rho correlations between the three EIBNF conditions in Experiment 2

Comparison	Correlation	95% confidence intervals
**Neutral–High-intensity**	.85^***^	.77, .90
**Neutral–Mid-intensity**	.85^***^	.78, .90
**High-intensity–Mid-intensity**	.85^***^	.79, .90

*Note*. *** indicates *p* < .001

Following this, we again used split-half analyses (Parsons, 2020) to estimate rank-order reliability of the EIBNF magnitudes (i.e., the difference scores) as well as the absolute scores. These reliability scores are shown in Table 7. The overall pattern of results for the EIBNF data is the same as that for the EIB data obtained in Experiment 1: Reliability was poor for the EIBNF magnitudes—that is, for the difference scores between the neutral and emotionally salient conditions, but good for the absolute scores (greater the recommended 0.7 for individual-difference research; Hedge et al., 2018).

**Table 7:** Reliability scores for the two EIB magnitudes and performance on the three distractor conditions in Experiment 2

Condition	Reliability	95% confidence interval
**High-intensity EIB**	.23	−.01 to .45
**Mid-intensity EIB**	.08	−.21 to .36
**Neutral distractor**	.90	.86 to .93
**High-intensity distractor**	.90	.87 to .93
**Mid-intensity distractor**	.92	.88 to .94

#### Relationship between EIB and DASS-21

DASS scores for the sample had a mean of 31.93 and standard deviation of 20.65. Given that the distributions were not normally distributed (skew and kurtosis *z*-scores >2.58), we used Spearman’s ρ. Overall, EIB magnitudes for neither the EIBNF conditions, nor the absolute conditions were significantly related to DASS-21 scores. That is, there were no significant correlations with DASS-21, with Spearman’s correlations of *ρ*(90) = .02, *p* = .850 [−.19, .23] for the high-intensity condition and *ρ*(90) < .01, *p* = .980 [−.20, .21] for the mid-intensity condition. In addition, absolute performance on the individual conditions were also not correlated with DASS-21 scores, *ρ*(90) = .04, *p* = .740 [−.17, .24] for neutral, *ρ*(90) = .04, *p* = .732 [−.17, .24] for high- and *ρ*(90) = .06, *p* = .583 [−.15, .26] for mid-intensity conditions.

### Discussion

The EIBNF paradigm was not successful in improving the reliability of EIB scores either for mid- or high-intensity stimuli.

## General discussion

The EIB paradigm has been used to measure the bias of temporal-based attention to emotionally salient stimuli. The evidence in the literature to date points to mixed evidence regarding whether there is a relationship between EIB scores and individual differences in the intensity of negative affect (Goodhew & Edwards, 2022b). One potential source of these inconsistencies in the literature is likely the measurement reliability of EIB, which, when explicitly examined, has not been found to meet recommended benchmark levels. Therefore, the goal of the present study was to attempt to improve the rank-order reliability of EIB scores.

This work draws on the pioneering work of Hedge and colleagues (Hedge et al., 2018) that highlights the intrinsic tension between experimental and individual-differences (correlational) research with respect to the effects of between-participant variance. Specifically, that between-participant variance in scores is harmful to the goals of the former and beneficial to the latter. We reasoned that existing paradigms for obtaining EIB scores have likely maximized the magnitude of the impairment following emotionally salient compared with emotionally neutral distractors at the group level, and this may have undermined their utility for individual difference design (Goodhew & Edwards, 2019). Therefore, in Experiment 1, we sought to improve the reliability of EIB scores by using less emotionally intense stimuli as distractors. This was designed to increase between-participant variability in the magnitude of the effect, thereby promoting reliability. The results did not support this idea, with split-half reliability for the EIB scores (i.e., the difference in performance between the neutral and emotionally salient conditions) having poor reliability. As expected, the reliability for the scores for the absolute (nondifference) conditions were much higher, especially for the neutral and mid-intensity conditions, with both being above the level required for individual differences research (Hedge et al., 2018). Scores for all three conditions were highly correlated with each other.

Given that by taking a difference score the variance that is shared between the two conditions that enter into that difference score is removed, we next hypothesized that the marked reduction in reliability in going from the absolute scores to the EIB difference scores was due to this shared variance capturing an important component of attentional control. That is, the processes that drove individual variation in performance for the neutral condition may also play a significant role in driving individual variation in the two emotionally salient conditions. EIB is typically quantified using the difference scores because it removes aspects of individual performance that are not related to the attentional bias (Goodhew & Edwards, 2022b). However, as pointed out by Hoffman and colleagues, the neutral (and emotionally salient) images used in EIB are physically distinct from the filler images, and this physical distinctiveness would lead to an attentional oddball effect (Baker et al., 2021; Hoffman et al., 2020; Santacroce et al., 2023). This means that all three conditions would have this oddball aspect, which could be driving attentional allocation to them, while the two emotionally salient conditions would have the additional emotional aspect. This common attentional-control component in the conditions could account for the high correlation between the neutral and emotionally salient conditions, and hence the marked reduction in the reliability of the EIB scores. That is, by taking the difference between the neutral and emotionally salient conditions a significant component of the individual differences in attention-driven performance is removed. Consequently, in Experiment 2 we developed a new experimental paradigm (EIBNF) that contained no filler images. The aim was to remove the attentional oddball pop-out effect caused by the distractor being distinct from the long sequence of homogeneous filler images. Note that while the EIBNF paradigm removes the oddball effect, there still exists physical differences between the distractors (both emotionally salient and neutral distractors) and the other images, so the physical salience of the distractor, as a cue, cannot be entirely removed. The pattern of results obtained with this EIBNF paradigm were the same as those obtained in Experiment 1: high reliability for the absolute scores, poor reliability for the EIBNF magnitudes (i.e., the difference scores), and high correlation between the conditions.

Thus, the results of the current study indicate that neither decreasing the intensity of the emotionally salient conditions nor reducing the oddball effect of the distractors relative to fillers in the stream resulted in split-half reliabilities of the EIB and EIBNF magnitudes that would be acceptable for individual-difference studies. However, the reliability of the absolute scores for the various conditions did meet acceptable standards. This indicates that whatever these conditions are measuring results in consistent differences between participants and that the loss of reliability in going from these absolute scores to the difference scores is due to the high correlation between the scores for the various conditions.

The reliabilities that we obtained in our study are similar to that obtained by Onie and Most (2017). Their reliabilities were 0.8 for the absolute negative scores and 0.4 for the difference scores at Lag 2. Our reliabilities for Experiment 1 (where the standard EIB paradigm was used) fell in the confidence interval range of 0.6 to 0.7 and 0.1 to 0.5, respectively, for the absolute and difference scores using the standard, high-intensity stimuli. While the reliabilities are similar in these two studies, it is important to keep in mind that the reliability obtained in a study is not constant for a given experimental paradigm. The reliability obtained across studies, in this case, using the EIB paradigm, will vary according to factors that differ across the studies. For example, the actual distractor images used and variations in the characteristics of the participants in the studies on factors like anxiety and attentional control that could influence how they respond to those images. With respect to these issues, it would be ideal to have each participant in the study rate the images on the relevant dimensions, like valence and arousal. Note though, that ratings for valence and arousal appear to be substantially consistent across individuals, whereas ratings for other dimensions, like motivational intensity, can vary markedly (Campbell et al., 2023, 2024).

How, then, are we to interpret the current results? There are four key aspects to our results that need to be considered: (1) Reliability for the standard EIB difference scores is lower than that required for individual-differences research; (2) the reliability of the absolute scores is higher and is in the range required for individual differences research; (3) the scores for the various conditions are highly correlated with each other; (4) increasing the emotional intensity of the distractors led to a significant decrease in performance. That is, target identification accuracy (i.e., task performance) was best in the neutral condition, intermediate in the mid-intensity condition, and worst in the high-intensity condition.

Taken as a whole, this means that it was not the case that scores for the three conditions were highly correlated because their performances were the same (i.e., because increasing emotional salience had no effect). Rather, performance across the conditions significantly differed. Further, despite those differences, the rank ordering across the different conditions remained consistent, and it was likely this consistency in the rank ordering of the absolute scores for the conditions that led to the poor reliability of the standard EIB difference scores.

There are several implications of our findings for using the EIB paradigm for individual differences research. First and foremost, the low reliability of the standard difference score means that it is not suitable for individual-difference research. However, the reliability of the absolute scores for the different distractor conditions are high enough to be used in individual-difference research. The second implication is that the high correlation between the distractor conditions means that it is likely that common process/es underpin performance on them. Given that the neutral condition did not include emotionally salient stimuli, the third implication is that whatever drives this consistency in rank ordering across the conditions is not specific to any form of attentional bias to emotionally salient stimuli. That is, if, as is likely, performance is being driven by attentional processes, the pattern of performance across participants does not selectively reflect attentional control in relation to emotionally salient stimuli, not even the emotionally salient conditions. That is, while adding emotional salience to the distractors impairs performance, it does not selectively reflect attentional biases to emotionally salient stimuli. This conclusion that EIB performance (even with the emotional distractors) reflects generic (i.e., nonemotionally salient specific) control is consistent with views of other authors who argue that it is only the physical salience of the emotional distractors that drives the greater attentional engagement with them, not the emotional salience of them (Baker et al., 2021; Santacroce et al., 2023). That is, both types of distractors (neutral and emotionally salient) are physically distinct from the filler images, and it is this physical salience that results in attentional engagement with them and hence impaired performance in those condition. Further, the emotionally salient distractors are more physically distinct than the neutral distractors, hence there is greater attentional engagement with them and hence even worse performance than with the neutral distractors (Baker et al., 2021; Santacroce et al., 2023).

However, an argument against this idea is that EIB can be achieved with stimuli that are otherwise neutral but that have been conditioned to be negative. That is, the EIB effect of physically identical stimuli can be made greater via learning that they predict negative or positive outcomes (i.e., associative learning). Importantly, the impairment with these stimuli occurs at multiple lags, including Lag 2 (Le Pelley et al., 2019; Smith et al., 2006). These findings argue against the effect of the emotionally salient distractor at Lag 2 being purely due to the physical characteristics of the distractor images. Taken as a whole, what these studies likely mean is that engagement with the distractor at Lag 2 can be driven by both the physical and emotional salience of the image. The relative contribution of the two would depend upon the actual images being used and how sensitive the participant is to them.

Given the issues identified above (specifically, the low reliability of the standard EIB difference scores and the potential for variation across studies due to the use of different images and participants), the mixed findings in the literature are not surprising. That said, as discussed previously, most studies have failed to find a relationship between EIB performance (using either the standard EIB difference score or absolute scores) and individual difference scores. Additionally, those researchers who have found such differences have failed to replicate those findings in subsequent studies (Guilbert et al., 2020; Kennedy et al., 2020; Kennedy & Most, 2015; Most et al., 2005, 2006; Onie & Most, 2017; Perone et al., 2020). Why then do some studies find these relationships? One possible explanation is Type 1 errors. That is, there really are not any relationships between these variables and chance has driven the occasional observation of them. However, another possibility is that, as mentioned above, these different results are due to variations in the images used and the characteristics of the participant samples used in the various studies. According to this possibility, there really is a relationship between these variables, but it is only observed under particular conditions, one of which may be sufficient reliability. While we cannot adjudicate between these two possibilities here, the present work highlights the importance of considering measurement reliability in considering such associations.

It has been suggested that negative affect factors such as trait anxiety may be linked to impaired domain-general attentional control, not limited to circumstances in which emotionally significant stimuli are present (Bishop, 2009; Eysenck et al., 2007; Moran, 2016; Shi et al., 2019). If absolute performance in both the emotional and neutral conditions in EIB reflect attentional control, then why was negative affect not correlated with these absolute scores in either experiment here? A recent meta-analysis suggests that the effects of trait anxiety may be particularly pronounced on efficiency measures of attentional control (e.g., reaction time), rather than effectiveness (e.g., accuracy; Shi et al., 2019). On the one hand, since EIB uses accuracy scores, it could be considered an effectiveness outcome. If so, then the lack of relationship between negative affect and performance in the current study is consistent with this framework. On the other hand, however, given that EIB is a temporally specific effect that arises from rapidly presented stimuli, it could be considered an efficiency measure. From this perspective, it is less clear why we did not observe a relationship between negative affect and performance. It could be because negative affect as gauged by the DASS asks about recent experiences of mood, which is conceptually distinct from trait anxiety, both in terms of not being a pure measure of trait negative affect, and negative affect being a broader construct than anxiety specifically. It would be interesting in future research to test whether negative affect correlates with more clear-cut efficiency metrics such as emotion-induced slowing.

There are a number of aspects of our study that potentially limit its generalizability. First, we used only a single lag (Lag 2). This means that our results are specific to this short (200 ms) time frame. We chose this lag because it typically gives a strong EIB effect. Of course, this means that using the standard high intensity negative distractors and a longer lag would be another way to potentially minimize the intensity of the EIB effect. Further, as stated above, it has been suggested that the emotional aspect of the distractor has an effect, not on the initial engagement with the distractor (which is arguably what Lag 2 is tapping) but the disengagement from it (which is arguably what a later lag would be tapping; Baker et al., 2021; Hoffman et al., 2020; Santacroce et al., 2023). If this is correct, then an interesting avenue for future research would be to apply the approaches used here to test reliability and relationships to individual-difference factors such as negative affect at longer lags. Again, though, the finding that conditioned stimuli produce a significant Lag 2 impairment argues that the emotional salience can play a role at Lag 2. Also, if there are differences in the time course of recovery (that is, individual differences in the magnitude of the EIB effect at longer lags) it would not be clear whether this was due to the effect of emotional salience on disengagement, or merely that it takes longer to recover from a greater impairment at Lag 2.

The second limiting factor of our study is that EIB scores gauge the temporal rather than the spatial allocation of attention. This means that any conclusions we can draw would be limited to temporal aspects of attention and may not generalize to spatial attention. Of course, ultimately attention works in an integrated manner, so examining how temporal and spatial aspects of attention work together may be particularly useful in future research. Dual-stream emotion-induced blindness in which two RSVP streams are spatially offset is one way that both the spatial and temporal aspects can be considered concurrently (Most & Wang, 2011; Proud et al., 2020). Interestingly, we have found a reliable association between trait anxiety and performance on that task (Proud et al., 2020). Third, our participant sample were composed of young adults (mean age for both studies was around 24 years). This means it is possible that different results could be obtained with an older sample. Older people tend to show a diminished EIB effect to negative distractors (Kennedy et al., 2020), and thus the reliability of EIB scores may be different in such a sample.

In addition, it is worth noting that our EIBNF experimental paradigm is a useful alternative to the standard EIB paradigm. It has the advantages of being quicker, due to having a shorter image sequence, which also makes it easier to implement when using online platforms. It can also be paired with our similar Emotion-Induced-Slowing (EIS) paradigm which uses a reaction-time measure (Goodhew & Edwards, 2022a) when the aim is to compare accuracy versus response-time measures.

A final observation concerns the possibility of using a true baseline condition as a reference. That is, to replace the neutral condition with one that does not contain any distractor, just another filler image (Jin et al., 2018). While this would remove the physical salience aspect from the reference condition, it would not do so for the emotionally salient condition. This means that the EIB effect would still be confounded by the physical salience aspect of the emotional distractor and there being no way to determine the relative magnitudes of the emotional and physical salience components.

## Conclusions

Here, our goal was to improve the reliability of EIB scores. We made two good-faith attempts to achieve this, but were not able to for the EIB difference scores. However, the absolute scores were reliable. But the strong correlations between the neutral and emotionally salient conditions highlighted that the rank ordering of individuals’ performance in the emotionally salient condition cannot be selectively attributed to emotional salience. Instead, they likely reflect generic attentional control demands shared with the neutral condition. These results revealed important insights about what is being measured in EIB, and potential avenues for future research.

## References

[CR1] Anderson, A. A. K. (2005). Affective influences on the attentional dynamics supporting awareness. *Journal of Experimental Psychology: General,**134*(2), 258–281. 10.1037/0096-3445.134.2.258 15869349 10.1037/0096-3445.134.2.258

[CR2] Anderson, B. A., Laurent, P. A., & Yantis, S. (2011). Value-driven attentional capture. *Proceedings of the National Academy of Sciences,**108*(25), 10367–10371. 10.1073/pnas.1104047108 10.1073/pnas.1104047108 PMC3121816 21646524

[CR3] Baker, A. L., Kim, M., & Hoffman, J. E. (2021). Searching for emotional salience. *Cognition,**214*, 104730. 10.1016/j.cognition.2021.104730 33975124 10.1016/j.cognition.2021.104730

[CR4] Bar-Haim, Y., Lamy, D., Pergamin, L., Bakermans-Kranenburg, M. J., & van Ijzendoorn, M. H. (2007). Threat-related attentional bias in anxious and nonanxious individuals: A meta-analytic study. *Psychological Bulletin,**133*(1), 1–24. 10.1037/0033-2909.133.1.1 17201568 10.1037/0033-2909.133.1.1

[CR5] Berggren, N., & Derakshan, N. (2013). Trait anxiety reduces implicit expectancy during target spatial probability cueing. *Emotion,**13*(2), 345–349. 10.1037/a0029981 23046457 10.1037/a0029981

[CR6] Bishop, S. J. (2009). Trait anxiety and impoverished prefrontal control of attention. *Nature Neuroscience,**12*(1), 92–98. 10.1038/nn.2242 19079249 10.1038/nn.2242

[CR7] Breitmeyer, B. G. (1984). *Visual masking: An integrative approach*. Clarendon Press.

[CR8] Campbell, N. M., Dawel, A., Edwards, M., & Goodhew, S. C. (2023). Motivational direction diverges from valence for sadness, anger, and amusement: A role for appraisals? *Emotion,**23*(5), 1334–1348. 10.1037/emo0001165 36074620 10.1037/emo0001165

[CR9] Campbell, N. M., Dawel, A., Edwards, M., & Goodhew, S. C. (2024). Four best-practice recommendations for improving the conceptualization and operationalization of motivational intensity: Reply to Kaczmarek and Harmon-Jones. *Emotion,**24*(1), 299–302. 10.1037/emo0001292 38227473 10.1037/emo0001292

[CR10] Carlson, J. M., & Fang, L. (2020). The stability and reliability of attentional bias measures in the dot-probe task: Evidence from both traditional mean bias scores and trial-level bias scores. *Motivation and Emotion. *10.1007/s11031-020-09834-6 10.1007/s11031-020-09834-6 PMC7864391 33551518

[CR11] Carrasco, M. (2011). Visual attention: The past 25 years. *Vision Research,**51*(13), 1484–1525. 10.1016/j.visres.2011.04.012 21549742 10.1016/j.visres.2011.04.012 PMC3390154

[CR12] Chapman, A., Devue, C., & Grimshaw, G. M. (2019). Fleeting reliability in the dot-probe task. *Psychological Research,**83*(2), 308–320. 10.1007/s00426-017-0947-6 29159699 10.1007/s00426-017-0947-6

[CR13] Chen, X., Duan, H., Kan, Y., Qi, S., & Hu, W. (2020). Influence of emotional task relevancy on the temporal dynamic of attentional bias in people with high-trait anxiety. *Journal of Cognitive Psychology,**32*(2), 242–253. 10.1080/20445911.2020.1719115

[CR14] Chong, S. C., & Treisman, A. (2005). Attentional spread in the statistical processing of visual displays. *Perception & Psychophysics,**67*(1), 1–13. 10.3758/bf03195009 15912869 10.3758/bf03195009

[CR15] Coltheart, M. (1980). Iconic memory and visible persistence. *Perception & Psychophysics,**27*(3), 183–228. 10.3758/bf03204258 6992093 10.3758/bf03204258

[CR16] Cox, J. A., Christensen, B. K., & Goodhew, S. C. (2018). Temporal dynamics of anxiety-related attentional bias: Is affective context a missing piece of the puzzle? *Cognition and Emotion,**32*(6), 1329–1338. 10.1080/02699931.2017.1386619 28984503 10.1080/02699931.2017.1386619

[CR17] Crawford, J. R., & Henry, J. D. (2003). The Depression Anxiety Stress Scales (DASS): Normative data and latent structure in a large non-clinical sample. *British Journal of Clinical Psychology,**42*(Pt 2), 111–131. 10.1348/014466503321903544 12828802 10.1348/014466503321903544

[CR18] Delchau, H. L., Christensen, B. K., Lipp, O. V., & Goodhew, S. C. (2022). The effect of social anxiety on top-down attentional orienting to emotional faces. *Emotion,**22*(3), 572–585. 10.1037/emo0000764 32478534 10.1037/emo0000764

[CR19] Edwards, J. R. (2001). Ten difference score myths. *Organizational Research Methods,**4*(3), 265–287. 10.1177/109442810143005

[CR20] Eysenck, M. W., Derakshan, N., Santos, R., & Calvo, M. G. (2007). Anxiety and cognitive performance: Attentional control theory. *Emotion,**7*(2), 336–353. 10.1037/1528-3542.7.2.336 17516812 10.1037/1528-3542.7.2.336

[CR21] Faul, F., Erdfelder, E., Buchner, A., & Lang, A. G. (2009). Statistical power analyses using G*Power 3.1: Tests for correlation and regression analyses. *Behavior Research Methods,**41*(4), 1149–1160. 10.3758/brm.41.4.1149 19897823 10.3758/BRM.41.4.1149

[CR22] Fox, E., Russo, R., & Dutton, K. (2002). Attentional bias for threat: Evidence for delayed disengagement from emotional faces. *Cognition and Emotion,**16*(3), 355–379. 10.1080/02699930143000527 18273395 10.1080/02699930143000527 PMC2241753

[CR23] Frewen, P. A., Dozois, D. J. A., Joanisse, M. F., & Neufeld, R. W. J. (2008). Selective attention to threat versus reward: Meta-analysis and neural-network modeling of the dot-probe task. *Clinical Psychology Review,**28*(2), 307–337. 10.1016/j.cpr.2007.05.006 17618023 10.1016/j.cpr.2007.05.006

[CR24] Frischen, A., Eastwood, J. D., & Smilek, D. (2008). Visual search for faces with emotional expressions. *Psychological Bulletin,**134*(5), 662–676. 10.1037/0033-2909.134.5.662 18729567 10.1037/0033-2909.134.5.662

[CR25] Geng, J., Won, B. Y., & Carlisle, N. (2019). Distractor ignoring: Strategies, learning, and passive filtering. *Current Directions in Psychological Scieence,**28*(6), 600–606. 10.1177/0963721419867099 10.1177/0963721419867099 PMC7983343 33758472

[CR26] Goodhew, S. C. (2020). *The breadth of visual attention* (J. T. Enns). Cambridge University Press. 10.1017/9781108854702

[CR27] Goodhew, S. C., & Edwards, M. (2019). Translating experimental paradigms into individual-differences research: Contributions, challenges, and practical recommendations. *Consciousness and Cognition,**69*, 14–25. 10.1016/j.concog.2019.01.008 30685513 10.1016/j.concog.2019.01.008

[CR28] Goodhew, S. C., & Edwards, M. (2022a). Both negative and positive task-irrelevant stimuli contract attentional breadth in individuals with high levels of negative affect. *Cognition and Emotion,**36*, 317–331. 10.1080/02699931.2021.2009445 34843423 10.1080/02699931.2021.2009445

[CR29] Goodhew, S. C., & Edwards, M. (2022b). Don’t look now! Emotion-induced blindness: The interplay between emotion and attention. *Attention, Perception, & Psychophysics,**84*(8), 2741–2761. 10.3758/s13414-022-02525-z 10.3758/s13414-022-02525-z PMC9630228 35701659

[CR30] Guilbert, D., Most, S. B., & Curby, K. M. (2020). Real world familiarity does not reduce susceptibility to emotional disruption of perception: evidence from two temporal attention tasks. *Cognition & Emotion,**34*(3), 450–461. 10.1080/02699931.2019.1637333 31282266 10.1080/02699931.2019.1637333

[CR31] Hedge, C., Powell, G., & Sumner, P. (2018). The reliability paradox: Why robust cognitive tasks do not produce reliable individual differences. *Behavior Research Methods,**50*(3), 1166–1186. 10.3758/s13428-017-0935-1 28726177 10.3758/s13428-017-0935-1 PMC5990556

[CR32] Henry, J. D., & Crawford, J. R. (2005). The short-form version of the Depression Anxiety Stress Scales (DASS-21): Construct validity and normative data in a large non-clinical sample. *British Journal of Clinical Psychology,**44*(Pt 2), 227–239. 10.1348/014466505x29657 16004657 10.1348/014466505X29657

[CR33] Hoffman, J. E., Kim, M., Taylor, M., & Holiday, K. (2020). Emotional capture during emotion-induced blindness is not automatic. *Cortex,**122*, 140–158. 10.1016/j.cortex.2019.03.013 31003713 10.1016/j.cortex.2019.03.013

[CR34] JASP Team. (2020). *JASP* (Version 0.14.1) [Computer software]. https://jasp-stats.org/

[CR35] Jin, M., Onie, S., Curby, K. M., & Most, S. B. (2018). Aversive images cause less perceptual interference among violent video game players: Evidence from emotion-induced blindness. *Visual Cognition,**26*(10), 753–763. 10.1080/13506285.2018.1553223

[CR36] Kappenman, E. S., MacNamara, A., & Proudfit, G. H. (2014). Electrocortical evidence for rapid allocation of attention to threat in the dot-probe task. *Social Cognitive and Affective Neuroscience,**10*(4), 577–583. 10.1093/scan/nsu098 25062842 10.1093/scan/nsu098 PMC4381248

[CR37] Kennedy, B. L., & Most, S. B. (2015). The rapid perceptual impact of emotional distractors. *PLOS ONE,**10*(6), e0129320. 10.1371/journal.pone.0129320 26075603 10.1371/journal.pone.0129320 PMC4468095

[CR38] Kennedy, B. L., Huang, R., & Mather, M. (2020). Age differences in emotion-induced blindness: Positivity effects in early attention. *Emotion,**20*(7), 1266–1278. 10.1037/emo0000643 31403807 10.1037/emo0000643 PMC7012746

[CR39] Kruijt, A. W., Parsons, S., & Fox, E. (2019). A meta-analysis of bias at baseline in RCTs of attention bias modification: No evidence for dot-probe bias towards threat in clinical anxiety and PTSD. *Journal of Abnormal Psychology,**128*(6), 563–573. 10.1037/abn0000406 31368735 10.1037/abn0000406

[CR40] Lang, P. J., Bradley, M. M., & Cuthbert, B. N. (2008). *International Affective Picture System (IAPS): Affective ratings of pictures and instruction manual (Tech. Rep. A-8)*. University of Florida.

[CR41] Le Pelley, M. E., Watson, P., Pearson, D., Abeywickrama, R. S., & Most, S. B. (2019). Winners and losers: Reward and punishment produce biases in temporal selection. *Journal of Experimental Psychology: Learning, Memory, and Cognition,**45*(5), 822–833. 10.1037/xlm0000612 29985032 10.1037/xlm0000612

[CR42] Lipp, O. V., & Derakshan, N. (2005). Attentional bias to pictures of fear-relevant animals in a dot probe task. *Emotion,**5*(3), 365–369. 10.1037/1528-3542.5.3.365 16187873 10.1037/1528-3542.5.3.365

[CR43] Lovidbond, S. H., & Lovibond, P. F. (1995). *Manual for the depression anxiety stress scales*. Psychology Foundation.

[CR44] MacLeod, C., Mathews, A., & Tata, P. (1986). Attentional bias in emotional disorders. *Journal of Abnormal Psychology,**95*(1), 15–20. 10.1037//0021-843x.95.1.15 3700842 10.1037//0021-843x.95.1.15

[CR45] MacLeod, C., Grafton, B., & Notebaert, L. (2019). Anxiety-linked attentional bias: Is it reliable? *Annual Review of Clinical Psychology,**15*(1), 529–554. 10.1146/annurev-clinpsy-050718-095505 30649926 10.1146/annurev-clinpsy-050718-095505

[CR46] Mogg, K., Bradley, B., Miles, F., & Dixon, R. (2004). Time course of attentional bias for threat scenes: Testing the vigilance-avoidance hypothesis. *Cognition and Emotion,**18*(5), 689–700. 10.1080/02699930341000158

[CR47] Mogg, K., Holmes, A., Garner, M., & Bradley, B. P. (2008). Effects of threat cues on attentional shifting, disengagement and response slowing in anxious individuals. *Behaviour research and therapy,**46*(5), 656–667. 10.1016/j.brat.2008.02.011 18395185 10.1016/j.brat.2008.02.011 PMC2862288

[CR48] Moran, T. P. (2016). Anxiety and working memory capacity: A meta-analysis and narrative review. *Psychological Bulletin,**142*(8), 831–864. 10.1037/bul0000051 26963369 10.1037/bul0000051

[CR49] Most, S. B., & Wang, L. (2011). Dissociating spatial attention and awareness in emotion-induced blindness. *Psychological Science,**22*(3), 300–305. 10.1177/0956797610397665 21270446 10.1177/0956797610397665

[CR50] Most, S. B., Chun, M. M., Widders, D. M., & Zald, D. H. (2005). Attentional rubbernecking: Cognitive control and personality in emotion-induced blindness. *Psychonomic Bulletin & Review,**12*(4), 654–661. 10.3758/BF03196754 16447378 10.3758/bf03196754

[CR51] Most, S. B., Chun, M. M., Johnson, M. R., & Kiehl, K. A. (2006). Attentional modulation of the amygdala varies with personality. *NeuroImage,**31*(2), 934–944. 10.1016/j.neuroimage.2005.12.031 16492400 10.1016/j.neuroimage.2005.12.031

[CR52] Most, S. B., Smith, S. D., Cooter, A. B., Levy, B. N., & Zald, D. H. (2007). The naked truth: Positive, arousing distractors impair rapid target perception. *Cognition and Emotion,**21*, 964–981. 10.1080/02699930600959340

[CR53] Onie, S., & Most, S. B. (2017). Two roads diverged: Distinct mechanisms of attentional bias differentially predict negative affect and persistent negative thought. *Emotion,**17*(5), 884–894. 10.1037/emo0000280 28230392 10.1037/emo0000280

[CR54] Parsons, S. (2020). Splithalf: Robust estimates of split half reliability. *The Journal of Open Source Software,**6*(60), 3. 10.21105/joss.03041

[CR55] Parsons, S., Kruijt, A.-W., & Fox, E. (2019). Psychological Science Needs a Standard Practice of Reporting the Reliability of Cognitive-Behavioral Measurements. *Advances in Methods and Practices in Psychological Science,**2*(4), 378–395. 10.1177/2515245919879695

[CR56] Pauli, W. M., & Röder, B. (2008). Emotional salience changes the focus of spatial attention. *Brain Research,**1214*, 94–104. 10.1016/j.brainres.2008.03.048 18466885 10.1016/j.brainres.2008.03.048

[CR57] Perone, P., Becker, D. V., & Tybur, J. M. (2020). Visual disgust elicitors produce an attentional blink independent of contextual and trait-level pathogen avoidance. *Emotion. *10.1037/emo0000751 10.1037/emo0000751 32324005

[CR58] Posner, M. I. (1980). Orienting of attention. *The Quarterly Journal of Experimental Psychology,**32*(1), 3–25. 10.1080/00335558008248231 7367577 10.1080/00335558008248231

[CR59] Proud, M., Goodhew, S. C., & Edwards, M. (2020). A vigilance avoidance account of spatial selectivity in dual-stream emotion induced blindness. *Psychonomic Bulletin & Review,**27*(2), 322–329. 10.3758/s13423-019-01690-x 31898265 10.3758/s13423-019-01690-x

[CR60] Santacroce, L. A., Swami, A. L., & Tamber-Rosenau, B. J. (2023). More than a feeling: The emotional attentional blink relies on non-emotional “pop out,” but is weak compared with the attentional blink. *Attention, Perception, & Psychophysics,**85*(4), 1034–1053. 10.3758/s13414-023-02677-6 10.3758/s13414-023-02677-6 PMC10014141 36918514

[CR61] Schmukle, S. C. (2005). Unreliability of the dot probe task. *European Journal of Personality,**19*(7), 595–605. 10.1002/per.554

[CR62] Shi, R., Sharpe, L., & Abbott, M. (2019). A meta-analysis of the relationship between anxiety and attentional control. *Clinical Psychology Review,**72*, 101754. 10.1016/j.cpr.2019.101754 31306935 10.1016/j.cpr.2019.101754

[CR63] Smith, S. D., Most, S. B., Newsome, L. A., & Zald, D. H. (2006). An emotion-induced attentional blink elicited by aversively conditioned stimuli. *Emotion,**6*(3), 523–527. 10.1037/1528-3542.6.3.523 16938093 10.1037/1528-3542.6.3.523

[CR64] Spearman, C. (1910). Correlation calculated from faulty data. *British Journal of Psychology 1904–1920,**3*(3), 271–295. 10.1111/j.2044-8295.1910.tb00206.x

[CR65] Sutherland, M. R., McQuiggan, D. A., Ryan, J. D., & Mather, M. (2017). Perceptual salience does not influence emotional arousal’s impairing effects on top-down attention. *Emotion,**17*(4), 700–706. 10.1037/emo0000245 28080087 10.1037/emo0000245 PMC5444951

[CR66] Tovée, M. J. (1998). *The speed of thought: Information processing in the cerebral cortex*. Springer. 10.1007/978-3-662-10408-8_4

[CR67] Vuilleumier, P., Armony, J. L., Clarke, K., Husain, M., Driver, J., & Dolan, R. J. (2002). Neural response to emotional faces with and without awareness: Event-related fMRI in a parietal patient with visual extinction and spatial neglect. *Neuropsychologia,**40*(12), 2156–2166. 10.1016/S0028-3932(02)00045-3 12208011 10.1016/s0028-3932(02)00045-3

[CR68] Zvielli, A., Bernstein, A., & Koster, E. H. W. (2015). Temporal dynamics of attentional bias. *Clinical Psychological Science,**3*(5), 772–788. 10.1177/2167702614551572

[CR69] Zvielli, A., Vrijsen, J. N., Koster, E. H., & Bernstein, A. (2016). Attentional bias temporal dynamics in remitted depression. *Journal of Abnormal Psychology,**125*(6), 768–776. 10.1037/abn0000190 27505407 10.1037/abn0000190

